# HiSeeker: Detecting High-Order SNP Interactions Based on Pairwise SNP Combinations

**DOI:** 10.3390/genes8060153

**Published:** 2017-05-31

**Authors:** Jie Liu, Guoxian Yu, Yuan Jiang, Jun Wang

**Affiliations:** College of Computer and Information Science, Southwest University, Chongqing 400715, China; jiel@email.swu.edu.cn (J.L.); gxyu@swu.edu.cn (G.Y.); yuanjiang@email.swu.edu.cn (Y.J.)

**Keywords:** genome-wide association studies, high-order SNP interactions, logistic regression model, ant colony optimization

## Abstract

Detecting single nucleotide polymorphisms’ (SNPs) interaction is one of the most popular approaches for explaining the missing heritability of common complex diseases in genome-wide association studies. Many methods have been proposed for SNP interaction detection, but most of them only focus on pairwise interactions and ignore high-order ones, which may also contribute to complex traits. Existing methods for high-order interaction detection can hardly handle genome-wide data and suffer from low detection power, due to the exponential growth of search space. In this paper, we proposed a flexible two-stage approach (called HiSeeker) to detect high-order interactions. In the screening stage, HiSeeker employs the chi-squared test and logistic regression model to efficiently obtain candidate pairwise combinations, which have intermediate or significant associations with the phenotype for interaction detection. In the search stage, two different strategies (exhaustive search and ant colony optimization-based search) are utilized to detect high-order interactions from candidate combinations. The experimental results on simulated datasets demonstrate that HiSeeker can more efficiently and effectively detect high-order interactions than related representative algorithms. On two real case-control datasets, HiSeeker also detects several significant high-order interactions, whose individual SNPs and pairwise interactions have no strong main effects or pairwise interaction effects, and these high-order interactions can hardly be identified by related algorithms.

## 1. Introduction

Genome-wide association studies (GWAS) have been widely used in complex disease study and have detected hundreds of single-nucleotide polymorphisms (SNPs) associated with complex diseases [1]. However, the identified single risk SNPs can only explain a portion of the theoretical estimated heritability of complex diseases [2,3,4]. That is partially because commonly-used univariate analysis techniques in GWAS can only be used to detect SNPs with strong marginal effects. Complex diseases are influenced by many genetic variants and environmental factors; nonlinear interaction effects of multiple SNPs may also uncover a portion of unexplained heritability of complex diseases [5,6,7]. Single locus-based methods may not detect these interactions, especially for those with small or little marginal effects, and thus, limit the success of GWAS for complex diseases [8]. To alleviate this limit, how to detect genome-wide interactions has been attracting more and more attention [5,9].

Two major challenges are faced with detecting SNP interactions on whole genome-scale data. The first challenge is the intensive computational burden caused by the “curse of dimensionality” and “combinatorial explosion” [5,10]. More than ten billion combinations need to be evaluated, even if only considering all possible pairwise SNP interactions. The other is the statistical challenge to achieve significance thresholds derived following Bonferroni correction of a large number of tests [5]. To combat these challenges, some computationally-efficient algorithms were proposed [5,11]. For example, the fast epistasis test implemented in PLINK [12] uses a classical logistic regression and odds-ratio contrast to infer epistasis. Wan et al. [13] proposed a Boolean operation-based screening and testing method, which designs a Boolean representation of genotype data and uses fast logic operations to obtain contingency tables to efficiently detect two-locus interactions from genome-wide datasets. Zhang et al. [14] developed an approach that utilizes a minimum spanning tree structure to update contingency tables without scanning all individuals for epistatic interaction detection.

Recent studies found that high-order interactions contribute to variability in complex traits for model organisms [15,16,17,18]. It is recognized that high-order interactions are critical in metabolic networks in yeast [16] and *Escherichia coli* [17]. Uncovered two-gene to four-gene interactions show different pleiotropic effects on branching and flowering in *Arabidopsis* [18]. In addition to that, high-order interactions may also contribute to the development of complex diseases in human beings. However, most of these aforementioned methods only focus on pairwise SNP interactions and ignore high-order interactions. Moreover, these high-order ones cannot be easily detected by standard two-locus tests. Some exhaustive methods can be extended to search high-order interactions, but they can only handle a relatively small number of SNPs (tens or hundreds). Collins et al. [19] utilized multifactor dimensionality reduction (MDR) [20] to identify statistically significant three-locus interactions that are associated with tuberculosis. Hu et al. [21] proposed a new measure based on information gain to detect three-locus interactions.

However, it is impractical to exhaustively search all high-order interactions on a genome-wide scale due to the exponential increase of the search space. Given a GWAS data with 1100K SNPs and 2000 samples, Goudey et al. [22] estimated that it requires more than five years to evaluate all three-way interactions on a highly parallelized computing server with about 262,000 cores. Thus, some stochastic methods [23,24,25] have been proposed to find some approximate optimal solutions. Wang et al. [23] developed a two-stage ant colony optimization algorithm called AntEpiSeeker to detect high-order interactions. Aflakparast et al. [24] combined a Bayesian scoring function with an evolutionary-based heuristic search to detect high-order interactions on grouped SNPs. Wang et al. [25] took advantage of Markov chain Monte Carlo search and a Bayesian computational method to detect high-order interactions on each chromosome or filtered SNPs. Some tree-based approaches have been proposed to search disease-associated joint associations with the consideration of high-order interactions [26]. Lu et al. [27] combined a likelihood ratio-based Mann–Whitney test and forward selection algorithm to search interactions among SNPs with moderate marginal effects. Wei et al. [28] proposed an ensemble method called tree assembling Mann–Whitney (TAMW), which combines many uncorrelated tree models to search interactions from SNPs with low marginal effects. Some two-stage methods have also been utilized to detect high-order interactions from significant pairwise interaction candidates, such as the epistasis detector based on the clustering of relatively frequent items (EDCF) [29], dynamic clustering for high-order genome-wide epistatic interactions detecting (DCHE) [30] and the co-information-based N-order epistasis detector and visualizer (CINOEDV) [31]. Both EDCF and DCHE adopt a stepwise search strategy and start with two-locus interaction models to detect significant high-order interactions on genome-wide data. The main difference between EDCF and DCHE is that EDCF partitions all genotype combinations of an SNP combination into three subgroups, whereas DCHE dynamically partitions genotype combinations into three to six subgroups and then utilizes the chi-squared test to evaluate a candidate combination based on its subgroup. CINOEDV firstly employs a co-information-based measure to detect two-locus combinations that have significant association with the phenotype, then builds a hyper-graph based on these combinations to visually discover high-order interactions.

These aforementioned methods have shown their abilities in detecting high-order interactions; they still have several limitations. Since these methods only concentrate on searching high-order interactions based on SNPs with strong main effects or significant two-locus interactions, high-order interactions may exist among risk loci with low or intermediate marginal effects, and pairwise interactions decomposed from these high-order interactions may not always be significant [11,25,27]. Most of these methods only take into account SNPs with strong marginal effects or significant pairwise interactions; thus, they may exclude some high-order interactions, in which the relevant SNPs and pairwise interactions between them have no significant effects.

**Figure 1:**
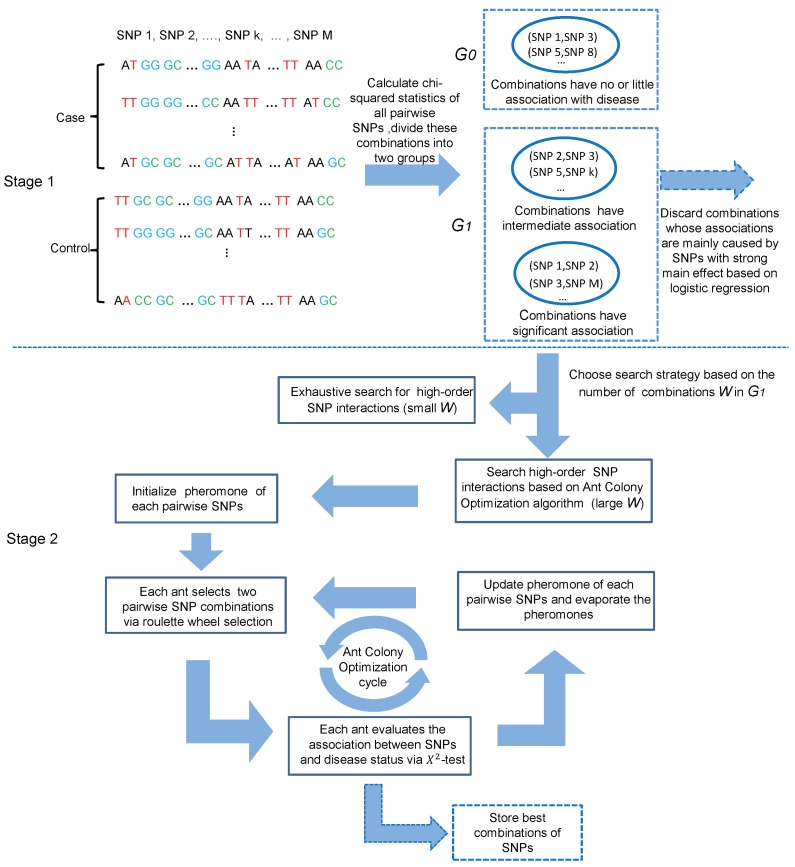
Framework of HiSeeker.

We proposed a two-stage (screening and searching) approach named HiSeeker to efficiently and effectively detect high-order interactions from pairwise combinations with strong or intermediate interaction effects. In the screening stage, the chi-squared test is firstly employed to quantify association effects of all pairwise combinations with the phenotype, and combinations with significant or intermediate association are retained based on the chi-squared test statistics. Next, HiSeeker resorts to logistic regression model to further reduce the cardinality of candidates by removing combinations whose associations with the phenotype are mainly caused by strong marginal effects. In the search stage, HiSeeker utilizes two different search strategies to detect high-order interactions from the candidate set. For a small set, HiSeeker uses exhaustive search. For a large set, since the candidate set still includes a large number of two-locus combinations, HiSeeker employs a heuristic search method based on ant colony optimization (ACO). The whole framework of HiSeeker is illustrated in Figure 1.

We performed extensive simulation studies on six high-order disease models and compared its power with other representative methods, including AntEpiSeeker [23], TAMW [28], EDCF [29] and DCHE [30]. HiSeeker shows efficient and better performance in detecting high-order interactions. Our experiments on two real case-control datasets, breast cancer (BC) data and Celiac disease (CD) data, demonstrate that HiSeeker is feasible for high-order interaction study on the genome-wide scale. HiSeeker detects several two-locus and three-locus interactions that are significantly associated with disease traits. Particularly, HiSeeker detects two three-locus interactions, in which all individual SNPs have no strong main effects, and pairwise combinations decomposed from them also have no strong interaction effects. These detected interactions indicate that the subsets of high-order interactions may not always be significant. In contrast, previous methods that search high-order interactions based on SNPs with strong marginal effects or significant pairwise combinations can hardly identify such interactions.

## 2. Materials and Methods

In this article, we primarily focus on case-control study and assume all of the SNPs are biallelic. Given the genotype data at *M* SNPs of *N* individuals with dichotomous disease status. Let $n_{u}$ and $n_{a}$ denote the number of normal individuals (i.e., controls) and the number of affected individuals (i.e., cases), respectively. We use $X_{i}$ to denote the *i*-th SNP, i=1,2,⋯,M and *Y* to denote the disease status (1 for case and 0 for control). We use capital letters (A, B) to denote major alleles and lowercase letters (a, b) to denote minor alleles. A genotype is encoded as 0, 1 or 2 according to the number of copies of minor allele present at each locus. Our method aims to identify *K*-locus (*K*≥ 3) interactions significantly associated with disease based on selected two-locus combinations. In the following, we elaborate on the process of HiSeeker illustrated in Figure 1.

### 2.1. Stage I: Valid Two-Locus Combination Candidate Selection

#### 2.1.1. Two-Locus Combination Filtering

**Figure 2:**
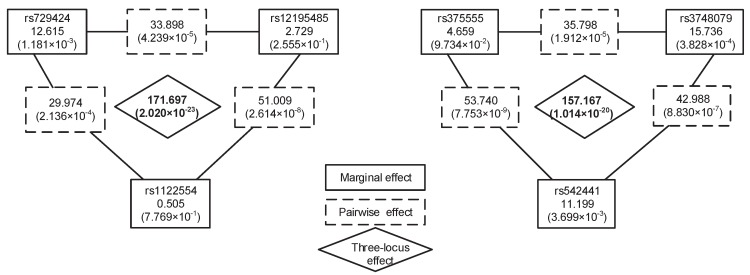
Two significant three-locus interactions identified by HiSeeker in the WTCCC CD data. A solid rectangle is an SNP with its name and marginal effect. A dashed rectangle represents the pairwise interaction effect of two SNPs. The diamond represents three-locus interaction effect. The marginal effect, pairwise effect and three-locus effect are evaluated by the chi-squared test. Values in brackets below the chi-squared statistics are the corresponding *p*-values. The Bonferroni-corrected significance levels for single-locus, two-locus and three-locus test are $1.181\times 10^{-7}$, $5.582\times 10^{-13}$, and $3.957\times 10^{-17}$, respectively.

Exhaustive analysis of all *K*-locus (*K*≥ 3) combinations is an intuitive solution for *K*-locus interaction detection. However, it is impractical to exhaustively search all *K*-locus interactions on a genome-wide scale due to the exponential increase of search space. Even so, some two-stage methods have been proposed to search high-order interactions, such as EDCF, DCHE and CINOEDV. These methods simply take top-*k* most significant two-locus interactions or two-locus interactions passing a specified significance threshold (i.e., Bonferroni-corrected significance level) as candidate interactions for high-order ones. However, pairwise interactions decomposed from high-order interactions may not always be significant. Here, we take two three-locus interactions in Figure 2 as the example to describe the case. These three-locus interactions are detected by our method in the Wellcome Trust Case-Control Consortium (WTCCC) CD data [32]. As Figure 2 shows, these six individual SNPs have no strong marginal effects, and these six pairwise interactions decomposed from the two three-locus interactions also have no significant associations with disease. However, these three-locus interactions have significant associations with the disease, even under a conservative Bonferroni-corrected significance level. These two stage methods mentioned above may lose their power to detect such interactions.

**Figure 3:**
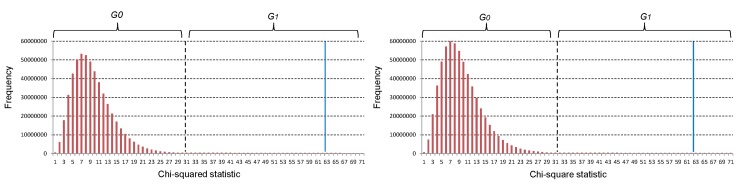
Distributions of chi-square statistics of all pairwise SNPs. The left and right histogram are the distribution of SNP combinations in Chromosomes 1 and 2 on the WTCCC CD data, respectively. Each bar indicates the number of pairwise combinations with the chi-square statistics in a continuous interval (1 in length), except the last bar, for which the interval is infinity. The blue solid line indicates the chi-squared statistic corresponding to a Bonferroni-corrected significance threshold. A combination whose chi-squared statistics is on the right of the blue solid line indicates that it has significant association with disease.

Besides considering combinations having significant interaction effects, we also take into account two-locus combinations having intermediate association with disease to obtain high-order interactions. For a genomic dataset, most two-locus combinations have no significant association with disease and are unlikely to be component interactions of high-order ones. Extensive statistical analysis on the distribution of two-locus combinations chi-square statistics on simulated datasets or a real GWAS dataset shows that most combinations have relatively small statistical test values and usually distribute in a relatively concentrated region (see Figure 3). The remaining combinations with intermediate or high statistical test values are distributed in a broader region. Studies on some other real GWAS datasets also show similar distributions [33]. It is helpful and feasible to identify high-order interactions from combinations with intermediate association with disease, since the number of these combinations is much smaller than the number of combinations with little or no association with disease. For example, two-locus combinations (rs12195485, rs1122554) and (rs375555, rs542441) have intermediate association with disease; they are considered by HiSeeker and found to be helpful for the identification of three-locus interactions.

Motivated by these observations, we partition two-locus combinations into two groups, denoted as $G_{0}$, $G_{1}$, where $G_{0}$ includes all two-locus combinations that have no or little association with disease, and $G_{1}$ includes combinations that have intermediate or significant association with disease. The chi-squared test is simple and powerful and can identify SNP combinations associated with disease without considering the disease model [23,34,35], so we resort to the chi-squared test to measure the associations between two-locus combinations and disease. Each two-locus combination is divided into one of these two groups according to its chi-squared statistic. To do such division, we need to derive some thresholds for declaring significance. The details of the setting threshold and grouping two-locus combinations are listed as follows:

- For given genotype data with *M* SNPs, to measure the association between the combination ($X_{i},X_{j})$ and disease, a contingency table like Table 1 is firstly constructed, then chi-squared statistic $\chi_{\left(X_{i},X_{j}\right)}^{2}$ is calculated as:
  (1) $$\chi_{\left(X_{i},X_{j}\right)}^{2}=\sum_{u=1}^{3^{2}}\sum_{v=1}^{2}\frac{{\left(n_{uv}-n_{u+}n_{+v}/N\right)}^{2}}{n_{u+}n_{+v}/N}$$
  where $\chi_{\left(X_{i},X_{j}\right)}^{2}$ follows a chi-squared distribution with 8 degrees of freedom. The chi-squared statistics of all two-locus combinations in *G* are denoted as $\chi_{d}^{2}\left(d=1,2,...,C_{M}^{2}\right)$.
- To obtain combinations having significant or intermediate association with disease, we firstly distinguish significant combinations from *G* that pass the Bonferroni correction. A combination with a statistic $\chi_{d}^{2}>\chi^{2}\left(\alpha \right)$ is placed into $G_{1}$, where $\chi^{2}\left(\alpha \right)$ denotes the corresponding chi-squared statistic of the Bonferroni-corrected significance level α. Given a preset significance level $\alpha_{0}$, α is calculated as:
  (2) $$\alpha =\alpha_{0}/C_{M}^{2}$$
- To obtain combinations having intermediate association with disease, a significance level $\alpha^{\prime }$ is defined as:
  (3) $$\alpha^{\prime }=\omega \alpha =\omega \alpha_{0}/C_{M}^{2}$$
  where ω≥1 is a scale factor that adjusts the number of combinations in $G_{1}$, which is retained for following analysis. The combinations with the chi-squared statistic $\chi_{d}^{2}$ between $\chi^{2}\left(\alpha^{\prime }\right)$ and $\chi^{2}\left(\alpha \right)$ have intermediate association with disease; they are also placed into $G_{1}$. All of the other combinations are placed into $G_{0}$.

The setting of ω considers the statistical distribution of all pairwise combinations and also takes into account that the Bonferroni correction is too conservative. $\alpha^{\prime }$ is adjusted by ω. A large ω corresponds to a lower significance level $\alpha^{\prime }$, and it can retain more two-locus combinations, which helps to retain more useful information and to identify more high-order interactions. On the other hand, more retained combinations ask for more runtime. The setting of ω should balance the dilemma between interaction loss and runtime cost.

#### 2.1.2. Candidate Combinations Screening

For large real GWAS datasets, our partition scheme based on the chi-squared test still produces a large number of candidate two-locus combinations for further analysis. Goudy et al. [33] utilized the chi-squared test and identified a large number significant two-locus combinations on three WTCCC datasets, even given the conservative Bonferroni-corrected significance level [33]. The number of significant pairwise interactions is more than 500,000 on Crohn’s disease, rheumatoid arthritis and type I diabetes datasets, and the number dramatically increases with the increase of SNPs with strong marginal effects. However, most of these significant pairs are not true epistatic interactions as their perceived association. That is due to the main effects [33,36]. Since two-locus combinations having intermediate association with disease are taken into account by HiSeeker, more noisy interactions are introduced for the next stage analysis, and the power of HiSeeker will downgrade.

To alleviate the negative influences of main effects, we employ the likelihood ratio test based on the logistic regression model [37] to screen the filtered two-locus combinations. For each SNP in $G_{1}$, a single-locus chi-squared test for the main effect is performed. If the corresponding *p*-value of an SNP is smaller than a Bonferroni-corrected significance threshold, it indicates that this SNP has a strong main effect. Next, to measure the association caused by main univariate effects only, a two-locus logistic regression model and the likelihood ratio test are utilized to evaluate the combinations with one or two SNPs with strong main effects in $G_{1}$. The details for measuring the association caused by main effects are listed as follows:

- For a two-locus combination ($X_{i},X_{j}$), HiSeeker first fits a full logistic regression model to measure the full association between ($X_{i},X_{j}$) and disease status *Y* (1 for case and 0 for control) as follows:
  (4) $$log\left(\frac{P(Y=1|\left(X_{i},X_{j}\right))}{P(Y=0|\left(X_{i},X_{j}\right))}\right)=\beta_{0}+\beta_{i}X_{i}+\beta_{j}X_{j}+\gamma X_{i}X_{j}$$
  where $\beta_{i}$ and $\beta_{j}$ are the main effects for SNP $X_{i}$ and $X_{j}$, respectively, and γ represents the interaction effect. Then, the Newton–Raphson method is utilized to iteratively optimize the corresponding maximum likelihood value ${\hat{L}}_{F}$ of Equation (4).
- For a two-locus combination, if both $X_{i}$ and $X_{j}$ have strong main effects, a logistic regression model defined by Equation (5) is fitted to measure the additive main effects of them as:
  (5) $$log\left(\frac{P(Y=1|\left(X_{i},X_{j}\right))}{P(Y=0|\left(X_{i},X_{j}\right))}\right)=\beta_{0}+\beta_{i}X_{i}+\beta_{j}X_{j}$$
  If only $X_{i}$ (or $X_{j}$) has a strong main effect, a logistic regression model defined by Equation (6) is used to measure the main effect in that combination as:
  (6) $$log\left(\frac{P(Y=1|\left(X_{i},X_{j}\right))}{P(Y=0|\left(X_{i},X_{j}\right))}\right)=\beta_{0}+\beta_{i}X_{i}$$
  Then, the Newton–Raphson method is utilized again to iteratively optimize the corresponding maximum likelihood value ${\hat{L}}_{M}$ of Equation (5) or Equation (6).
- HiSeeker calculates the deviation *D* of each two-locus combination in $G_{1}$ as follows:
  (7) $$D=2(\ln{\hat{L}}_{F}-\ln{\hat{L}}_{M})$$
  where *D* follows a chi-squared distribution with degree of freedom df. df=4 if both $X_{i}$ and $X_{j}$ have strong marginal effect; df=6 if only $X_{i}$ (or $X_{j}$) has strong main effect.

A small deviation indicates that the association between the combination and disease is mainly caused by SNPs with strong main effect. If the corresponding *p*-value of a combination’s deviation is larger than the threshold $\alpha^{\prime }$, this combination is discarded by HiSeeker; otherwise, it is retained for next stage analysis. To guarantee the efficiency, HiSeeker does not use the likelihood ratio test on a dataset, in which no strong marginal effect exists.

### 2.2. Stage 2: High-Order SNP Interaction Detection

In the second step, HiSeeker provides two types of search strategies to detect high-order interactions from candidate two-locus combinations obtained in the first step. These two-locus combination candidates are denoted as $C_{i}\left(i=1,2,...,W\right)$, where *W* denotes the number of candidates.

#### 2.2.1. Exhaustive Search Strategy for Small Candidate Set (Small *W*)

Exhaustive analysis has more of a chance to identify high-order interactions, and it is feasible to exhaustively analyze all high-order interactions when the number of candidates is small. Thus, we utilize the exhaustively search strategy on candidate combination sets with a small cardinality. To exhaustively search *K*-SNP (*K* ≥ 3) high-order interactions, we merge all of the SNPs in *W* combination candidates together and denote the merged SNP set as *S*. Then, all of the *K*-SNP combinations in *S* are evaluated by the chi-squared test to calculate corresponding *p*-values. HiSeeker reports the combinations whose *p*-values are smaller than a Bonferroni-corrected significance threshold.

#### 2.2.2. Ant Colony Optimization Strategy for Large Candidate Set (Large *W*)

When a large number of two-locus combinations is chosen in the first stage, it is very time consuming (or even infeasible) for these methods (i.e., EDCF and DCHE) to exhaustively search high-order interactions based on these combinations. Thus, different from EDCF and DCHE that only utilize the exhaustive search strategy, HiSeeker resorts to a swarm intelligence optimization algorithm named ant colony optimization (ACO) [38] to efficiently detect high-order interactions from a large number of candidate combinations.

ACO is a successful technique for a non-deterministic polynomial-time hard (NP-hard) combinatorial optimization problem; it has been widely used in GWAS studies [23,39,40]. Basically, the ACO-based method aims to search combinations that can clearly discriminate between the control and case samples within a GWAS dataset. The search space comprises all SNPs and their combinations. The power of ACO-based methods has been shown in detecting two-locus interactions on genome-wide datasets [23,40]. However, these stochastic approaches based on ACO significantly lose their power in detecting high-order interactions. That is because the search space is exponentially increased, and ACO can hardly obtain optimal solutions through positive feedbacks. To reduce the negative effect of the large search space and help ACO obtain the optimal solution, we narrow the search space as the two-locus combinations chosen in the first stage.

Here, we use *K* = 3 as an example to detect *K*-SNP (K≥3) interactions. The ACO-based search strategy is listed as follows:

(i) Initialization: the pheromone value of each two-locus combination is initialized as a fixed value $\tau_{0}$, which means that the association between a combination and disease is treated with equal possibility. To identify possible candidate combinations to assemble the high-order SNP interaction sets, ACO iteratively selects and evaluates SNP combinations from *W* candidates via the following Step ii to Step iv, until a preset number of iterations is reached.
(ii) Combination selection: ACO introduces *n* operators called ants to select SNP combinations. *n* is set based on the candidate size *W* (n<W). In each iteration, an ant chooses *d* (d∈[2,W]) combinations as its targeted two-locus combination set. *d* is set according to the order number needed by users. To detect three-locus interactions, *d* is initially set as 2. The probability for an ant *x* (0≤x≤n) selecting a two-locus combination $C_{i}$ based on roulette wheel selection can be defined as:
  (8) $$p_{x}\left(i\right)=\frac{\tau_{i}^{\delta }\eta_{i}^{\beta }}{\sum_{j=1}^{W}\tau_{j}^{\delta }\eta_{j}^{\beta }}$$
  where $\tau_{i}$ is the pheromone value of $C_{i}$ and $\eta_{i}$ is the prior information on $C_{i}$. δ and β are parameters to determine the weight of pheromone value and the weight of prior information on each combination, respectively. Here, η, β and δ are set to 1, indicating that each combination is treated equally before the optimization phase.
(iii) Evaluation on the selected combinations: the statistic of the chi-squared test is applied as the fitness function. In each iteration, two selected combinations of each ant are merged into a new combination $C^{\prime }$. The fitness of $C^{\prime }$ is calculated using Equation (1) and denoted as $\chi_{C^{\prime }}^{2}$. If the number of SNPs in $C^{\prime }$ is 3 (or 4), $\chi_{C^{\prime }}^{2}$ follows a chi-squared distribution with degree of freedom $df=3^{3}-1$ (or $df=3^{4}-1$). Given the same significance level $\alpha_{0}$, the Bonferroni-corrected significance level is $\alpha_{0}/C_{M}^{3}$ (or $\alpha_{0}/C_{M}^{4}$) for the three-locus (or four-locus) combination. The corresponding chi-square statistic is $\chi^{2}\left(\alpha_{0}/C_{M}^{4}\right)$ (or $\chi^{2}\left(\alpha_{0}/C_{M}^{4}\right)$). $\chi^{2}\left(\alpha_{0}/C_{M}^{4}\right)$ is about two-times $\chi^{2}\left(\alpha_{0}/C_{M}^{3}\right)$. Thus, to avoid the loss of significant three-locus combinations, $\chi_{C^{\prime }}^{2}$ is multiplied with a scale factor fs when the number of SNPs in $C^{\prime }$ is 3. For HiSeeker, fs=2. In each iteration, the merged SNP combinations with the highest chi-squared statistics are stored.
(iv) Pheromone update: in each iteration, after the selected *d* two-locus combinations of each ant have been evaluated, the corresponding pheromone of each two-locus combination in an ant is updated as:
  (9) $$\tau_{i}=\left(1-\rho \right)\tau_{i}+\Delta \tau_{i}$$
  where ρ is the evaporating coefficient and $\Delta \tau_{i}$ is the changing pheromone value of the *i*-th two-locus combination $C_{i}$, which equals 0.01$\chi_{C^{\prime }}^{2}$ of this ant. This update process is repeated for all ants.

After applying ACO, the merged SNP combinations with the highest chi-squared statistics are reported. Next, HiSeeker utilizes the chi-squared test again to analyze the *K*-SNP subsets of all of these reported combinations and finally takes *K*-SNP subsets with a *p*-value below the Bonferroni-corrected significance level $\alpha_{0}/C_{M}^{K}$ as the detected *K*-SNP interactions.

## 3. Results

In this section, we evaluate the performance of HiSeeker on both simulated and real datasets in detecting high-order SNP interactions. In the simulation study, we compare HiSeeker with four recently-proposed approaches under several high-order interaction models. For the real case-control study, we apply HiSeeker on two real GWAS datasets, BC data and CD data.

### 3.1. Experiments on Simulated Datasets

To assess the performance of HiSeeker, we perform extensive simulation experiments using six different disease models and compare its power with four representative approaches: AntEpiSeeker [23], EDCF [29], DCHE [30] and TAMW [28]. We adopt the same measure of power proposed by Wan et al. [13] as follows:

(10) $$Power=\frac{S}{N_{D}}$$

where *S* is the number of datasets in which true interaction loci are successfully identified among all generated $N_{D}$ datasets. The reason for choosing the above four approaches is that these approaches have shown their ability for high-order interaction detection under several disease models. We do not compare with other popular methods, such as epiMODE [41] and SNPRuler [42], since EDCF shows more power than them [29]. Besides these four approaches, to verify the effectiveness of searching high-order interaction from two-locus combinations that have strong or intermediate association with disease, we also compare HiSeeker with the method ChiSq(S), which utilizes the chi-squared test to exhaustively search high-order interaction SNPs only from the significant two-locus combinations. In the following experiments, we use HiSeeker(E) (HiSeeker(A)) to represent our method that utilizes exhaustive search strategy (ACO search strategy). To study the effectiveness of the two search strategies adopted by HiSeeker, we separately compare HiSeeker(E) and HiSeeker(A) with other methods on the simulated data. In SNP interaction detection, it is difficult to identify disease loci that have no marginal effect. To evaluate the power of HiSeeker on detecting high-order interactions under different cases, we consider two types of disease models: disease models with/without a marginal effect in the simulation experiments.

#### 3.1.1. Case 1: Disease Loci with Marginal Effects

Although there are many two-locus interaction models employed in the GWAS study, only a few high-order interaction models have been proposed for the GWAS study. Here, we use four models with marginal effects to comparatively evaluate the effectiveness of these methods in detecting high-order interactions. Model 1 and Model 2 define three-locus interactions with a multiplicative effect and a threshold effect, which are the extension of two well-known two-locus interaction models in Marchini et al. [9]. Models 3 and 4 are proposed by Zhang et al. [43]. Model 3 contains a three-locus interaction, and the marginal effect of each disease locus in Model 3 ranges from zero to a very small value. Model 4 has a six-locus interaction, and the interaction effect size of this model is controlled by θ, which is set to 50, the same as in Zhang et al. [43]. The definition of the marginal effect size λ of a disease locus is:

(11) $$\lambda =\frac{P_{Aa}/\left(1-P_{Aa}\right)}{P_{AA}/\left(1-P_{AA}\right)}-1$$

where $P_{AA}$ and $P_{Aa}$ denote the penetrance of genotype AA and Aa, respectively. The marginal effect size in simulation experiments is relatively small, λ = 0.2 for Model 1 and λ = 0.3 for Models 2 and 3. We set the marker minor allele frequencies (MAFs) of the disease loci as 0.1, 0.2 and 0.4. To evaluate the impact of linkage disequilibrium (LD) between disease loci and associated markers (measured by $r^{2}$) on the performance of these methods, we consider two scenarios: $r^{2}$ = 1 is simulated for directly genotyped disease loci; $r^{2}$ = 0.7 is simulated for disease loci ungenotyped, but their LD markers with $r^{2}$ = 0.7 genotyped. We use the same simulation program in BEAM [43] to simulate 100 datasets under each setting for each disease model, and each dataset contains 1000 SNPs. To take into account sample size *N*, we simulate 3000 samples and 6000 samples with balanced design (i.e., $n_{a}=n_{u}=N/2$).

**Figure 4:**
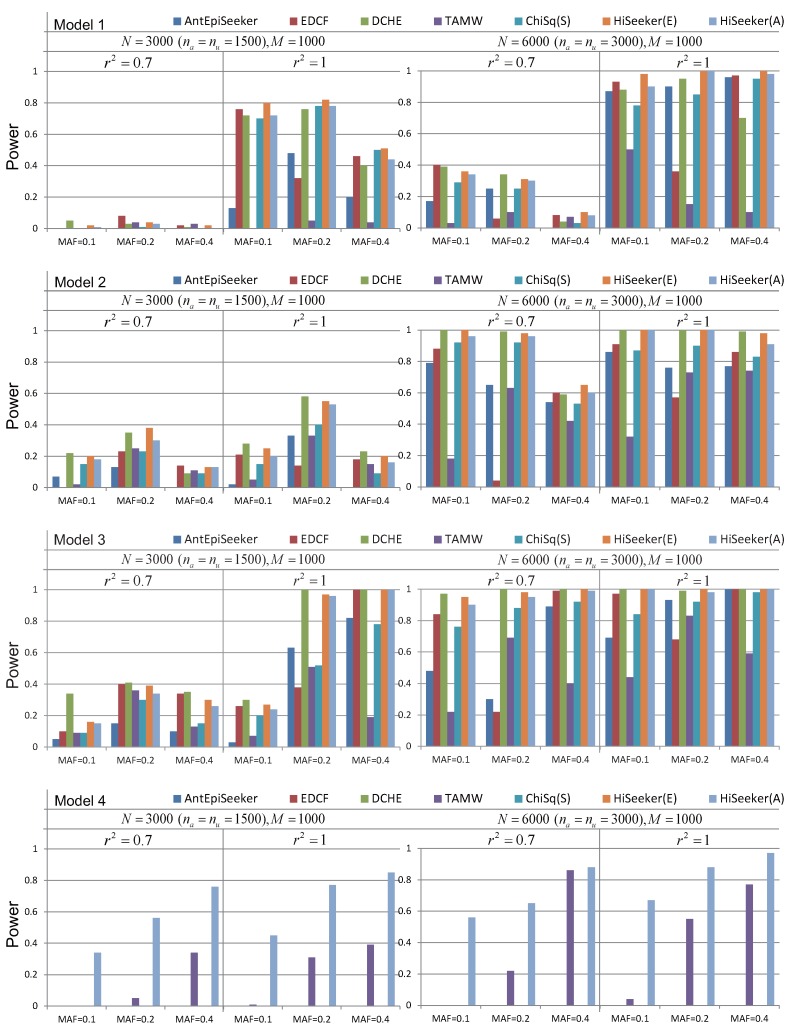
Powers of AntEpiSeeker, EDCF, DCHE, TAMW, ChiSq(S), HiSeeker(E) and HiSeeker(A) on four disease models with different allele frequencies and sample sizes. $n_{a}$ and $n_{u}$ denote the number of cases and controls, respectively. The absence of a bar indicates no power.

Figure 4 reveals the performance of different methods on these four models with marginal effects. As shown in Figure 4, the power of all of these methods increases with the growth of the sample size. The performance of these methods in scenario $r^{2}$ = 1 is better than in scenario $r^{2}$ = 0.7. Since Models 1 and 2 are the extension of multiplicative and threshold models, the power of most methods decreases when the MAF of the disease loci varies from 0.2 to 0.4; the trend is consistent with the results in Marchini et al. [9] and Wan et al. [13]. Since more valid candidates are retained by HiSeeker(E), HiSeeker(E) has better performance than other methods on the three-locus models (Models 1 to 3), except when the sample size is small. In such a case, HiSeeker(E) has lower power than DCHE. That is because DCHE measures significance via the chi-squared test with lower degrees of freedom than HiSeeker(E); DCHE can report more interactions in this case. HiSeeker(E) performs better than ChiSq(S); this comparison proves that high-order SNP interactions can be derived from the two-locus combinations with intermediate association with disease. TAMW has the lowest power in most cases, since it utilizes a forward selection algorithm, and at least one of the selected loci must have a reasonably strong marginal effect. Another interesting observation is that when the MAF of disease loci is 0.2, the power of EDCF drastically decreases. One possible reason is that EDCF divides each three-locus combination into three groups and uses the chi-squared test with two degrees of freedom to measure the significance, resulting in more false positives. Exhaustively searching hundreds of pairwise combinations for six-way interactions is still very time-consuming. Since some of these comparing methods do not provide the corresponding parameters for the six-way interaction detection, the performance of DCHE, EDCF, AntEpiSeeker and HiSeeker(E) is not compared for Model 4. The results of TAMW and HiSeeker(A) for Model 4 demonstrate that the stochastic search based on pairwise combinations is an alternative for high-order interaction detection.

HiSeeker(E) has better performance than two ACO-based methods AntEpiSeeker and HiSeeker(A), since exhaustive search is guaranteed to find optimal solutions without considering the time complexity. Although both AntEpiSeeker and HiSeeker(A) utilize the ACO algorithm, HiSeeker(A) has relatively superior performance compared to AntEpiSeeker. That is because HiSeeker(A) searches high-order SNP interactions from a pruned space and removes a large number of noisy combinations. HiSeeker(A) has comparable power with EDCF for detecting high-order interactions in most cases. These results also demonstrate the effeteness of HiSeeker(A) in detecting high-order interactions on small datasets.

#### 3.1.2. Case 2: Disease Loci without Marginal Effects

A pure interaction model is usually defined by the penetrance table, whose elements represent the probability of being affected with the disease given the genotype combination. The penetrance values are usually decided by three parameters: disease prevalence (P(D)), genetic heritability ($h^{2}$) [44] and MAF. Here, we consider two three-locus pure interaction models. Model 5 is proposed by Culverhouse et al. [44], which yields maximum genetic heritability $h^{2}$ with no marginal effect for the population penetrance p∈(0,1/16] with MAF = 0.5. When the MAF of disease loci is set to 0.5 in Model 3, the loci will have no marginal effect, so we use Model 6 to denote the special case of Model 3. Heritability $h^{2}$ controls the phenotypic variation of these two models; it ranges from 0.01 to 0.4 and MAF is set to 0.5. We use the software GAMETES_2.0 [45] to generate 100 datasets under different settings for two pure interaction models, in which the sample size *N* ($n_{a}=n_{u}=N/2$) varies from 200 to 400 and the SNP size is fixed as 1000.

**Figure 5:**
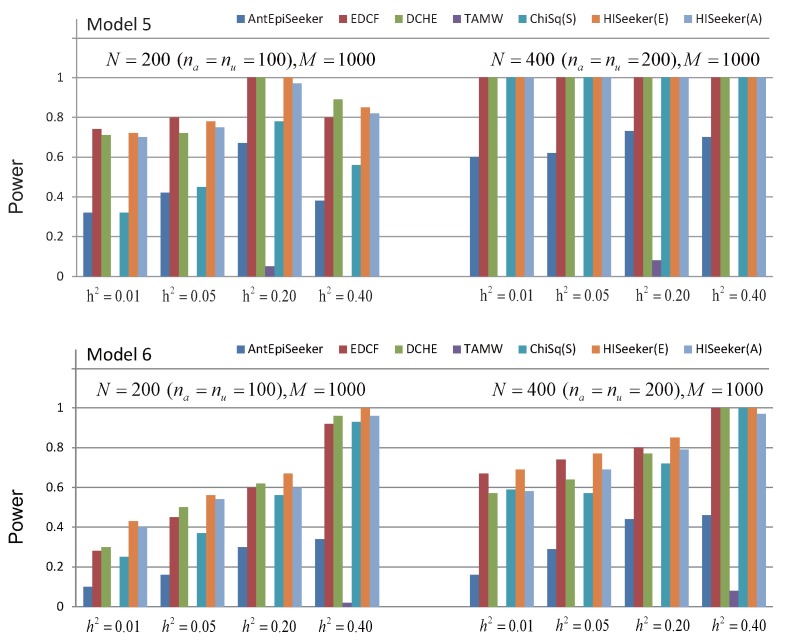
Powers of AntEpiSeeker, EDCF, DCHE, TAMW, ChiSq(S), HiSeeker(E) and HiSeeker(A) on two disease models without a marginal effect under different genetic heritabilities and sample sizes. The absence of a bar indicates no power.

Figure 5 shows the performance of these methods for high-order interaction detection on two models without marginal effect. For these two models, the power of all methods increases as the growth of heritability and sample size in most cases. For Model 5, all of the methods, except AntEpiSeeker and TAMW, have the same power when heritability ranges from 0.01 to 0.40 with the sample size as 400. The most probable reason is that pairwise interactions decomposed from three-locus interactions are always significant in such a case. TAMW loses its power to search interactions whose individual SNPs have no marginal effect. For these two models without marginal effects, the methods that search high-order interactions among pairwise combinations have better performance than AntEpiSeeker and TAMW. These results also demonstrate that HiSeeker can identify high-order interactions when there is no marginal effect.

### 3.2. Experiments on Real Datasets

#### 3.2.1. Experiments on BC Data

BC is the most common cancer in women. It is reported that breast cancer is caused by a combination of genetic and environmental risk factors [46]. We firstly applied HiSeeker on the BC dataset from the WTCCC project [47] to detect high-order interactions. This dataset contains genotypes of 15,347 SNPs from 1045 affected individuals and 2073 controls. Quality control is performed to exclude very low call rate samples and SNPs. An SNP is excluded if its call rate <95% across all samples, or its *p*-value (Hardy–Weinberg equilibrium) <0.0001 in controls. Samples are excluded for call rate <98%. After that, SNPs with MAF <0.1 are further excluded. After the quality control, the BC dataset contains 1045 case samples and 2070 control samples with 5607 SNPs.

HiSeeker takes 15 min to analyze these data on a server with Intel Xeon E5-2678, 256 GB RAM and CentOS 6.5. After the first stage (screening stage), 4151 two-locus combinations are retained for next stage analysis; among them there are several significant two-locus combinations (some representative ones are listed in Table 2). rs1108842 is located in gene *GNL3* on Chromosome 3. The protein encoded by *GNL3* may interact with p53 and may be involved in tumorigenesis. The encoded protein also appears to be important for stem cell proliferation. rs3785181 is in gene *GAS11*. *GAS11* includes 11 exons spanning 25 kb and maps to a region of Chromosome 16, and it is reported as being associated with BC [48].

In the search stage, HiSeeker identifies a significant three-locus combination (rs879882, rs2523608, rs592229) in the major histocompatibility complex (MHC) region on Chromosome 6, whose unadjusted *p*-value is 1.453E-33. rs3785181 is in gene *POU5F1*, which encodes a transcription factor containing a POU homeodomain that plays a key role in embryonic development and stem cell pluripotency [49]. Aberrant expression of this gene in adult tissues is associated with tumorigenesis. rs2523608 is located at gene *HLA-B*. *HLA-B* belongs to the human leukocyte antigen (HLA) class I heavy chain paralogs, which play a central role in the immune system. HLA class I antigen expression is closely related to the aggressiveness and prognosis of BC [50].

#### 3.2.2. Experiments on CD Data

CD is a common heritable chronic inflammatory condition of the small intestine induced by dietary wheat, rye and barley, as well as other unidentified environmental factors, in susceptible individuals [51]. The genome-wide CD dataset comprises 528,969 SNPs, 3796 cases and 8154 controls. Before applying HiSeeker on the CD dataset, the same quality control for BC dataset has also been applied to the CD dataset. Subsequently, 423,234 SNPs from 8154 controls and 3796 cases remained.

HiSeeker is applied to CD data on a server equipped with two Intel Xeon E5-2678 CPUs, 256 GB RAM and CentOS 6.5, where each core runs two threads. The exhaustive analysis for all two-locus combinations takes about 30 h; the follow-up ACO-based search for high-order interactions takes about 1.5 h. In the first stage, more than ten million two-locus combinations are chosen by the chi-squared test, and most of them contain one or two SNPs with strong marginal effect. After applying the likelihood ratio test based on logistic regression model, 28,451 two-locus combinations are retained for next stage analysis; among them, there are hundreds of significant two-locus combinations (some representative ones are listed in Table 3). Most of these significant two-locus combinations are located in the MHC region. rs210138 is located in gene *BAK1*. The protein encoded by *BAK1* belongs to the BCL2 protein family. BCL2 family members form oligomers (or heterodimers) and act as anti- or pro-apoptotic regulators that are involved in a wide variety of cellular activities. *BAK1* is confirmed in [52] to have strong association with CD. rs9262495 is in gene *DDX39B* (*BAT1*), which encodes an RNA helicase known to regulate the expression of *TNF* and *IL*-6. Elevated levels of these two cytokines are associated with increased severity of clinical outcomes [53].

In the second stage, HiSeeker(A) is utilized to detect high-order SNP interactions from the selected two-locus combinations. Two significant three-locus combinations are detected: (rs729424, rs12195485, rs1122554) and (rs375555, rs3748079, rs542441), whose unadjusted *p*-values are $2.020\times 10^{-23}$ and $1.014\times 10^{-20}$, respectively. They are also located in the MHC region on Chromosome 6. rs729424 is located in gene *ITPR3*, which encodes a receptor for inositol 1,4,5-trisphosphate, a second messenger that mediates the release of intracellular calcium [54]. rs542441 is in gene *UQCC2*, which encodes a nucleoid protein localized at the mitochondria inner membrane. The encoded protein affects the regulation of insulin secretion, mitochondrial ATP production and myogenesis through modulation of mitochondrial respiratory chain activity [55].

**Figure 6:**
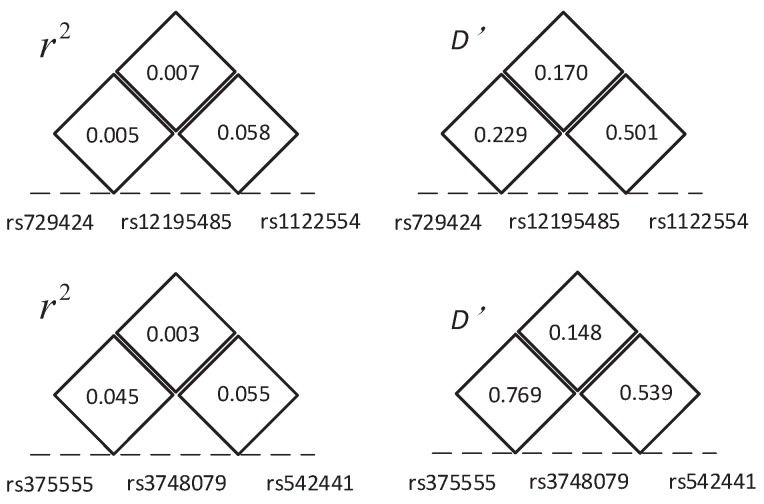
The linkage disequilibrium (LD) between SNPs in the two three-locus combination detected in CD data.

Furthermore, we utilized permutation test to evaluate the significance of these two combinations. In 10,000 permutation tests, both the *p*-values of these combinations are 0.0001. These *p*-values are smaller than a significance level of 0.05 and indicate those combinations having significant association with CD. The LD between these SNPs in each combination is shown in Figure 6; the small $r^{2}$ indicates these SNPs in low LD and also indicates that these interactions are not due to LD. The chi-squared statistics, corresponding *p*-values of the two combinations and their lower-order combinations are shown in Figure 2. As Figure 2 shows, all three single SNPs and three pairwise SNP combinations in each three-locus combination have no significant association with disease after Bonferroni correction for multiple tests. These results demonstrate that strong high-order SNP interaction can exist among risk loci with low or intermediate marginal effect, and lower-order interactions decomposed from them are not always significant. In contrast, the methods (i.e., TAMW and DCHE) that only focus on SNPs with strong marginal effects or significant pairwise SNP interactions can hardly detect such interactions.

### 3.3. Parameter Setting

In the screening stage of HiSeeker, ω adjusts the number of combinations retained for high-order interaction detection, and ω is set according to the number of SNPs *M*. A large ω retains more combinations with intermediate effects. For simulated and real datasets, we set $\omega =10^{4}$. In the search stage, when the number of candidate two-locus combinations *W* is small ($W<2\times 10^{3}$), exhaustive search strategy (HiSeeker(E)) is utilized to search all possible high-order interactions. For large *W* ($W>2\times 10^{3}$), the ACO-based search strategy (HiSeeker(A)) is utilized. There are five parameters in HiSeeker(A), including initial pheromone value $\tau_{i}$, the number of ants *n*, the evaporating coefficient ρ, the number of two-locus combinations *d* selected by an ant and the maximum number of iterations *MaxIter*. We specified these parameters according to previous studies of Wang et al. [23] and Jing et al. [35].

- $\tau_{i}$ of each two-locus combination is always set to 100, which means that we treat the association between each combination and disease with equal probability.
- For *K*-locus interaction detection, *d* should be set bigger than K/2. In the simulation study, we set d=2 for three-locus interaction detection and set d=3 for six-locus interaction detection.
- ρ ranges from 0.01 to 0.1 according to the number of candidate two-locus combinations *W*. A large ρ should be adopted for a small *W*. In the simulation study, we set ρ = 0.05. In the real study, ρ is set to 0.01.
- Both *n* and *MaxIter* are determined by *W*. We set *MaxIter* = 0.1 *W*. *n* ranges from 500 to 5000. A large *W* prefers large *n* and *MaxIter*.

### 3.4. Runtime Analysis

**Figure 7:**
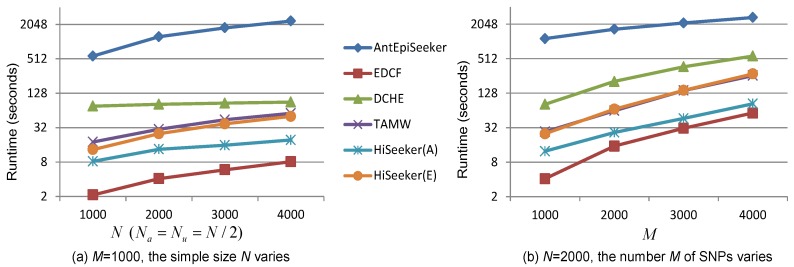
Runtime of different methods on simulated datasets. (**a**) The sample size *N* varies from 1000 to 4000 while the number of SNPs M=1000; (**b**) the number of SNPs varies from 1000 to 4000 while N=2000.

Computational efficiency is another key performance index that needs to be considered in detecting high-order interactions from genome-wide data. We compare the runtime of these comparing methods in detecting three-locus interactions by varying the sample size *N* and the number of SNPs *M*. All experiments are conducted on a server with Intel Xeon E5-2678, 256 GB RAM and CentOS 6.5. The recorded runtime of these methods is shown in Figure 7. Figure 7 shows that given a fixed number of SNPs, the runtime of all methods linearly increases with the increase of the sample size. Although EDCF, DCHE and HiSeeker exhaustively analyze all two-locus combinations, their runtimes are smaller than AntEpiSeeker. That is because EDCF, HiSeeker(A) and HiSeeker(E) utilize bitwise computing and store SNP genotype data in a bitwise data structure to achieve great memory efficiency and computing speed [13]. TAMW is also computationally efficient by employing forward selection algorithm. HiSeeker(A) is faster than HiSeeker(E), since exhaustive search is time consuming. EDCF detects high-order SNP interactions only based on selected significant two-locus combinations, whose number is much smaller than that used by HiSeeker, which additionally takes into account two-locus combinations that have intermediate association with disease. Therefore, EDCF is faster than HiSeeker.

## 4. Conclusions

In this paper, we proposed a two-stage method called HiSeeker to detect high-order SNP interactions from genome-wide case-control data. In the screening stage, HiSeeker first utilizes the chi-squared test to measure the association effects of all pairwise combinations with disease and retains combinations having significant or intermediate association with disease as candidates. Then, the likelihood test based on the logistic regression model is employed to further analyze these candidates, and the combinations whose association with disease are mainly caused by strong marginal effects are excluded. This screening mechanism can significantly reduce the search space for the following high-order interaction detection. In the search stage, two alternative search strategies, exhaustive search and ACO-based search, are provide by HiSeeker to detect high-order interactions from filtered candidate two-locus combinations. Exhaustive search is used for small candidate sets, and ACO-based search is used for large candidate sets. The flexible search mechanism can balance the efficiency and effectiveness of high-order interaction detection. Extensive experiments on simulated datasets illustrate that HiSeeker is more powerful than the other four recently-proposed methods. HiSeeker detected several significant high-order interactions on two real WTCCC datasets (breast cancer and Celiac disease). These examples demonstrate that it is feasible for HiSeeker to identify high-order interaction from genome-wide data. Particularly, HiSeeker found two three-locus combinations that have significant association with Celiac disease. Neither the three relevant loci have a strong main effect, nor do the three two-locus combinations have a significant interaction effect. This fact also validates that the high-order interaction can be originated from lower-order interactions without significant effects. We want to remind that existing methods only search high-order interactions from SNPs with strong marginal effects or significant pairwise interactions, and they can hardly identify such interactions.

HiSeeker can be an effective alternative to existing methods for detecting high-order interactions, and it displays several advantages over existing methods:

- HiSeeker flexibly screens two-locus combinations with strong or intermediate association with disease phenotype. This flexibility enables it to detect more high-order interactions, whose decomposed pairwise interactions are not significant.
- HiSeeker is not sensitive to the marginal effects of individual SNPs; since it makes use of the likelihood ratio test based on logistic regression to filter out the two-locus combinations, whose associations with disease are mainly caused by the SNPs with strong main effects.
- HiSeeker provides two alternative search strategies for datasets with different scales, and it enables detecting high-order interaction on large GWAS data without exhaustive enumeration.

Although HiSeeker shows good performance on both simulated and real datasets, it still suffers from the confounding factors; for example, haplotype effects, LD, missing genotype combinations, population stratification and others [7], which are also faced by AntEpiSeeker, EDCF, DCHE and TAMW. In future work, we will try to address these confounders as part of quality control procedures or after initial interaction tests. In addition, it is time consuming for high-order interaction detection. Therefore, we also plan to implement HiSeeker with graphics processing units (GPUs) to speedup HiSeeker.

## Figures and Tables

**Table 1:** A contingency table for the k-order interaction.

Combination	SNP 1	SNP 2	...	SNP k	Case	Control	Total
1	AA	BB		KK	$n_{11}$	$n_{12}$	$n_{1+}$
2	Aa	BB		KK	$n_{21}$	$n_{22}$	$n_{2+}$
3	aa	BB		KK	$n_{31}$	$n_{32}$	$n_{3+}$
:	:	:	:	:	:	:	:
:	:	:	:	:	:	:	:
$3^{k}-1$	aa	bb		Kk	:	:	:
$3^{k}$	aa	bb		kk	$n_{3^{k}1}$	$n_{3^{k}2}$	$n_{3^{k}+}$
Total					$n_{+1}$	$n_{+2}$	*N*

$n_{uv}$ means the number of samples with the *u*-th joint genotype for each SNP combination and the *v*-th disease status.

**Table 2:** Significant two-locus and three-locus combinations identified by HiSeeker on WTCCC BC data.

Significant Combination	Chromosome and Related Genes	Single-Locus *p*-Value	Combination *p*-Value
(rs1108842, rs4687657)	(chr3: GNL3, chr3: ITIH4)	($7.095\times 10^{-1}$, $7.302\times 10^{-1}$)	$2.541\times 10^{-143}$
(rs4408545, rs3785181)	(chr16: AFG3L1P, chr16: GAS11)	($3.666\times 10^{-1}$, $9.371\times 10^{-1}$)	$5.373\times 10^{-36}$
(rs3811040, rs2723192)	(chr2: CKAP2L, chr2: IL37)	($3.253\times 10^{-1}$, $2.011\times 10^{-1}$)	$1.329\times 10^{-29}$
(rs9379968, rs204994)	(chr6: *, chr6: AGER)	($2.229\times 10^{-3}$, $7.267\times 10^{-2}$)	$5.306\times 10^{-17}$
(rs9257694, rs3129943)	(chr6: OR14J1, chr6: LOC101929163)	($2.083\times 10^{-1}$, $5.486\times 10^{-1}$)	$5.201\times 10^{-16}$
(rs879882, rs2523608, rs592229)	(chr6:POU5F1, chr6:HLA-B, chr6:SKIV2L)	($1.532\times 10^{-1}$, $8.984\times 10^{-1}$, $6.503\times 10^{-2}$)	$1.453\times 10^{-33}$

* Indicates that the related gene is unknown. All of the *p*-values are not adjusted.

**Table 3:** Significant two-locus and three-locus combination identified by HiSeeker on WTCCC CD data.

Significant Combination	Chromosome and Related Genes	Single-Locus *p*-Value	Combination *p*-Value
(rs2844509, rs9262495)	(chr6: DDX39B, chr6: MUC22)	$4.448\times 10^{-2}$, $2.450\times 10^{-1}$)	$4.071\times 10^{-22}$
(rs2256028, rs406936)	(chr6: MICA, chr6: SKIV2L)	($1.529\times 10^{-1}$, $7.995\times 10^{-2}$)	$5.473\times 10^{-23}$
(rs210138, rs2894342)	(chr6: BAK1, Chr6:*)	($2.599\times 10^{-1}$, $2.011\times 10^{-1}$)	$1.566\times 10^{-19}$
(rs3130785, rs2844509)	(chr6: LINC00243, chr6: DDX39B)	($4.521\times 10^{-5}$, $4.448\times 10^{-2}$)	$8.182\times 10^{-16}$
(rs1519643, rs1481417)	(chr2: *, chr14:*)	($4.352\times 10^{-4}$, $2.175\times 10^{-6}$)	$1.663\times 10^{-13}$
(rs729424, rs12195485, rs1122554)	(chr6:ITPR3, chr6:LOC105375025, chr:*)	($1.181\times 10^{-3}$, $2.555\times 10^{-1}$, $7.769\times 10^{-1}$)	$2.020\times 10^{-23}$
(rs375555, rs3748079, rs542441)	(chr6: *, chr6: ITPR3, chr6: UQCC2)	($9.734\times 10^{-2}$, $3.828\times 10^{-4}$, $3.699\times 10^{-3}$)	$1.014\times 10^{-20}$

* Indicates that the related gene is unknown. All of the *p*-values are not adjusted.
